# Identification of key potential infection processes and risk factors in the computed tomography examination process by FMEA method under COVID-19

**DOI:** 10.1186/s12879-024-09136-z

**Published:** 2024-02-23

**Authors:** Lingzhi Jin, Meiting Ye, Wenhua Lin, Yong Ye, Yen-Ching Chuang, Jin-Yan Luo, Fuqin Tang

**Affiliations:** 1 Radiology department, Taizhou Central Hospital (Taizhou University Hospital), Taizhou, Zhejiang China; 2 Institute of Public Health and Emergency Management, Taizhou University, Taizhou, Zhejiang China; 3 Business College, Taizhou University, Taizhou, Zhejiang China; 4 Key Laboratory of evidence-based Radiology of Taizhou, Linhai, Zhejiang China; 5 Institute for Hospital Management, Tsing Hua University, Shenzhen, Guangdong China; 6 Nursing Department, Taizhou Central Hospital (Taizhou University Hospital), Taizhou, China

**Keywords:** Computed tomography, Failure modes and effects Analysis, Radiology, Risk analysis, Healthcare worker

## Abstract

**Purpose:**

To identify the key infection processes and risk factors in Computed Tomography (CT) examination process within the standard prevention and control measures for the COVID-19 epidemic, aiming to mitigate cross-infection occurrences in the hospital.

**Method:**

The case hospital has assembled a team of 30 experts specialized in CT examination. Based on the CT examination process, the potential failure modes were assessed from the perspective of severity (*S*), occurrence probability (*O*), and detectability (*D*); they were then combined with corresponding risk prevention measures. Finally, key infection processes and risk factors were identified according to the risk priority number (*RPN*) and expert analysis.

**Results:**

Through the application of RPN and further analysis, four key potential infection processes were identified, including “CT request form (*A*_1_),” “during the scan of CT patient (*B*_2_),” “CT room and objects disposal (*C*_2_),” and “medical waste (garbage) disposal (*C*_3_)”. In addition, eight key risk factors were also identified, including “cleaning personnel does not wear masks normatively (*C*_32_),” “nurse does not select the vein well, resulting in extravasation of the peripheral vein for enhanced CT (*B*_25_),” “patient cannot find the CT room (*A*_13_),” “patient has obtained a CT request form but does not know the procedure (*A*_12_),” “patient is too unwell to continue with the CT scan (*B*_24_),” “auxiliary staff (or technician) does not have a good grasp of the sterilization and disinfection standards (*C*_21_),” “auxiliary staff (or technician) does not sterilize the CT machine thoroughly (*C*_22_),” and “cleaning personnel lacks of knowledge of COVID-19 prevention and control (*C*_33_)”.

**Conclusion:**

Hospitals can publicize the precautions regarding CT examination through various channels, reducing the incidence of CT examination failure. Hospitals’ cleaning services are usually outsourced, and the educational background of the staff employed in these services is generally not high. Therefore, during training and communication, it is more necessary to provide a series of scope and training programs that are aligned with their understanding level. The model developed in this study effectively identifies the key infection prevention process and critical risk factors, enhancing the safety of medical staff and patients. This has significant research implications for the potential epidemic of major infectious diseases.

## Introduction

Hospitals were challenged related to the transmission risk of SARS-CoV-2 between healthcare workers and patients during the COVID-19 outbreak [1]. For example, as of February 11, 2022, a statistical study indicated that 3,019 medical personnel from 422 Chinese hospitals providing diagnosis and treatment for patients with novel coronavirus pneumonia had contracted the virus [2]. A study conducted in a Dutch hospital revealed that healthcare workers played a predominant role in transmitting nosocomial infection, both in single and multi-person wards [1]. Furthermore, research demonstrates that healthcare workers constitute the most frequently infected group with covid-19. In addition, another study highlighted that the nosocomial infection of SARS-CoV-2 mainly resulted from close contact between medical staff and patients [3].

Since the outbreak of novel coronavirus pneumonia, diagnostic imaging has provided valuable radiological support for disease diagnosis and prognosis prediction, such as X-ray, and computed tomography (CT) examinations [2, 4]. Radiology departments are at high risk of nosocomial COVID-19 infection [5]. Radiology personnel engage in direct contact with patients and bear the dual pressures of infection prevention and control and radiation protection measures [2]. Radiology staff must work in fever clinics, infection and respiratory clinics, isolation wards, and other workplaces where they have close contact with patients with fever, suspected or confirmed cases, thereby exposing themselves to a high risk of infection [2]. Several studies have indicated that risk assessment and associated preventive measures, such as risk assessment, testing, symptom monitoring, and prevention guidelines, are essential for preventing COVID-19 nosocomial infection transmission [6, 7]. Therefore, establishing a radiology risk assessment model with covid-19 characteristics and identifying key potential risk areas and factors will help mitigate infection risk during the diagnostic radiation process, ensuring the safety of both radiology staff and patients.

Limited studies have combined quantitative risk methods with the analysis of nosocomial infections during COVID-19. For example, Hsiung, Tung [8] combine Multi-Criteria Decision-Making (MCDM) and Failure Mode and Effects Analysis (FMEA) to propose a comprehensive risk assessment model. This model facilitates a thorough risk analysis and explores the ranking associated with COVID-19 in hospital screening procedures. Thomas and Suresh [9] combined a multi-grade fuzzy approach with importance-performance analysis (IPA) to develop an assessment framework for Covid-19 prevention and protection measures in hospitals. This framework serves as a continuous assessment tool to enhance improve Covid-19 prevention operations. Kannangara, Seetulsingh [10] used root cause analyses to analyze COVID-19 cases to identify potential causes, routes of transmission, and areas for improvement in managing the COVID-19 outbreak within acute admission units. Meziane, Taous [11] used healthcare failure mode and effect analysis to identify potential failure modes, determine key risk factors, and define the mitigation measures to mitigate the risk of COVID-19 infection in the operating room. Few hospitals have used failure mode and effect analysis to study nosocomial infections during COVID-19, and no study has examined COVID-19 infection prevention and control within the context of the CT examination process using failure mode and effect analysis.

This study uses FMEA to establish a complete risk analysis model of the CT examination process to make up for the gap in this study. Further, standardize the operation process of radiological medical staff to improve the risk prevention and control of radiological infection in hospitals in the face of major infectious epidemics in the future and ensure the safety of medical staff and patients.

## Methods and materials

### Research design and analysis process

**Fig. 1:**
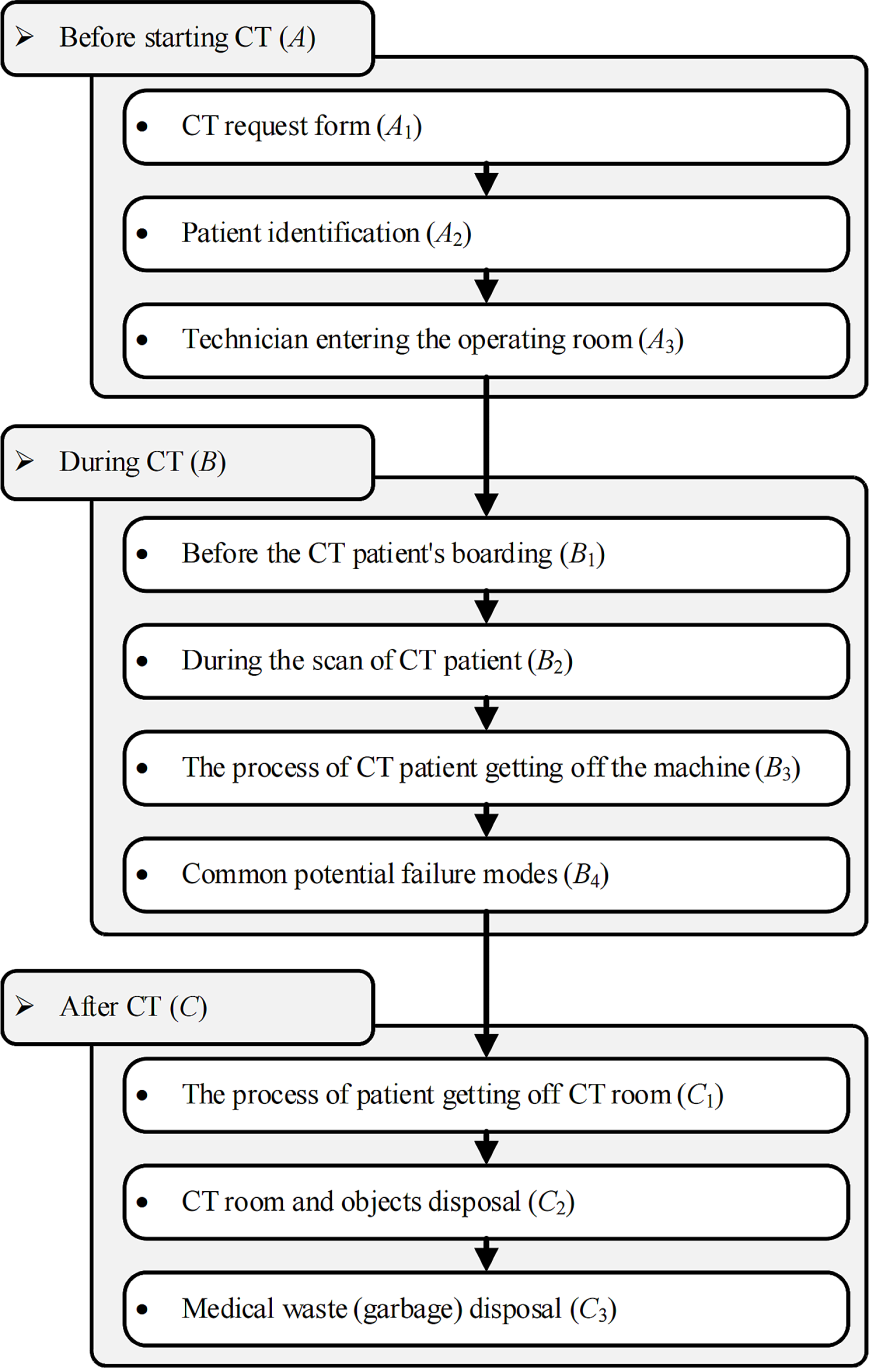
CT examination processes

**Fig. 2:**
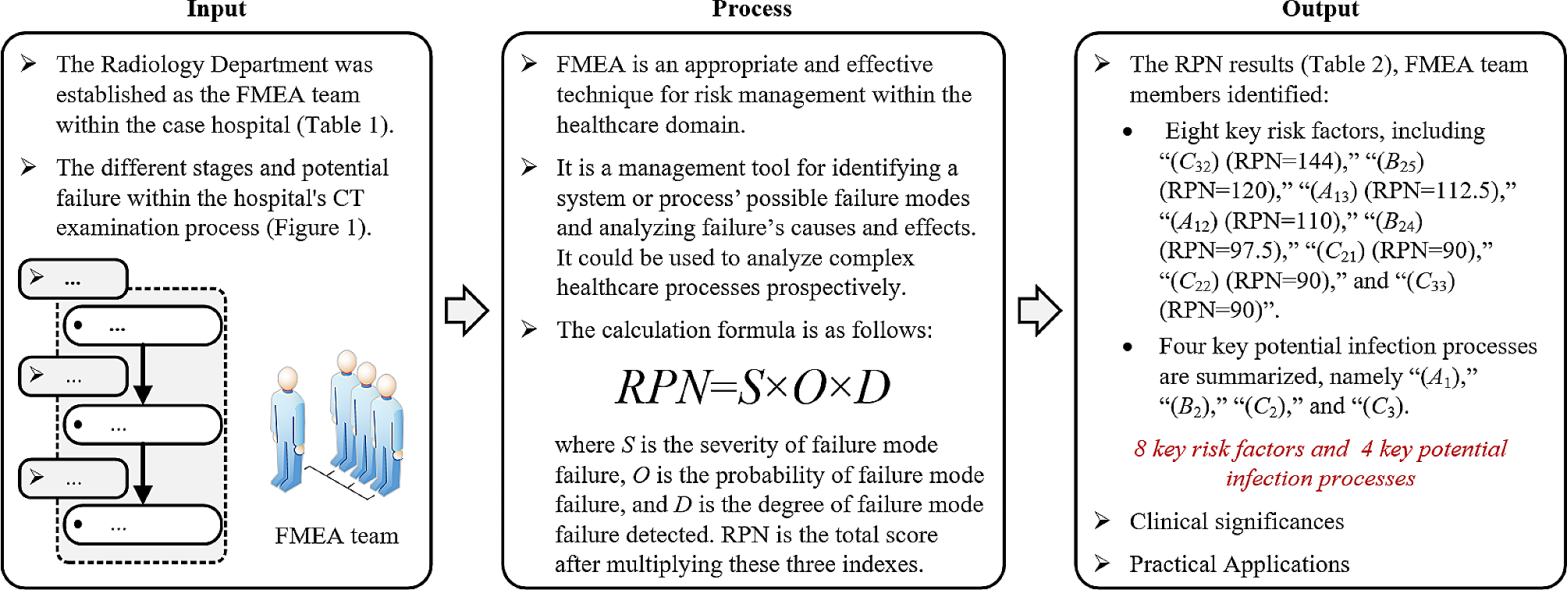
The research design and implementation process

The Radiology Department was established as the FMEA team within the case hospital. Their primary objective was to confirm the different stages and potential failure within the hospital’s CT examination process. They identified the stages of the CT process and the steps involved in each stage, as shown in Fig. 1, and summarized the potential failure modes during each stage according to relevant elements. Subsequently, they evaluated the potential failure patterns regarding severity (*S*), occurrence probability (*O*), and detectability (*D*), resulting in the determination of the risk priority number (*RPN*) of each stage. Finally, based on the RPN results and corresponding guidelines, the team determined key potential infection processes and risk factors in the CT examination process of the case hospital. The specific implementation process is shown in Fig. 2.

### FMEA

As a proactive risk management tool, FMEA is an appropriate and effective technique for risk management within the healthcare domain [12, 13]. It is a management tool for identifying a system or process’ possible failure modes and analyzing failure’s causes and effects [14]. It could be used to analyze complex healthcare processes prospectively.

The most important feature of FMEA is its capacity for proactive system failure prevention. It involves identifying, prioritizing, and addressing known or potential system failure modes before they occur [15, 16]. Presently, owing to its efficacy, it can reduce the occurrence of adverse events and reduce unnecessary costs. Nowadays, owing to its effectiveness, FMEA has been widely used in various fields, including medical [17, 18], manufacturing [19], aviation [20], electronics [21], the chemical industry [22], and other fields to evaluate system safety. In addition, FMEA has a wide range of applications for healthcare risk analysis, such as healthcare process, hospital management, hospital informatization, and medical equipment/production [14]. FMEA has mainly been applied to the healthcare processes, such as blood transfusion [23], medication use [24], radiation therapy [25], and other treatment processes, for quality improvement. Therefore, FMEA has high practicality for healthcare quality improvement and error reduction and has been employed to improve healthcare processes in hospitals.

The calculation formula is as follows: $RPN=S \times O \times D$ (1)

where *S* is the severity of failure mode failure, *O* is the probability of failure mode failure, and *D* is the degree of failure mode failure detected. RPN is the total score after multiplying these three indexes (i.e., *S*, *O*, and *D*), and the higher the value, the better the improvement.

### Ethical approval

The study’s design and procedure were conducted under the guidance of the Institute’s Ethics Review Committee and according to the principles of the Helsinki Declaration. This project has received ethical approval with the number 2023 L-01-03. All participant information remained anonymous.

### Data collection and participants

The case hospital assembled a multidisciplinary FMEA team comprising 30 experts from fields including Radiology, Emergency, Nursing, Medical, Hospitalization service center, Infectious disease, and Public health. The team was focused on the CT examination process and was through purposive sampling. Most of these team members are men (63%), aged 31 and 50 (70%). Most people have undergraduate education (77%) and over 6 years of work experience (94%). During the COVID-19 epidemic, all team members actively engaged in the clinical work within the hospital. Among them, 87% have worked in hospitals for over 31 months since the outbreak began and have had direct exposure to suspected or confirmed COVID-19 patients. The demographic information of domain experts is detailed in Table 1.

**Table 1:** Demographic information of the FMEA team for in-hospital CT examination

Types	Number	%
Sex		
Male	19	63
Female	11	37
Age		
Under 24 years old	2	7
25–30	2	7
31–40	9	30
41–50	12	40
51 ~ 60	5	16
Education level		
Junior College	5	17
Undergraduate	23	77
Master	1	3
Doctor	1	3
Work position		
Clinical medicine	11	37
Clinical nursing	7	23
Hospital management	2	6
Infection prevention and control	5	17
Other	5	17
Work department		
Radiology department	17	57
Emergency department	2	7
Nursing department	2	7
Medical department	3	10
Hospitalization service center	1	3
Infectious disease department	4	13
Public health department	1	3
Working experience		
Less than 5 years	2	6
6 to 10 years	5	17
11 to 20 years	8	27
21 to 30 years	8	27
More than 30 years	7	23
Whether to participate in hospital work during the epidemic		
Yes	30	100
No	0	0
Time spent participating in hospital work during the epidemic		
7 to 12 months	1	3
13 to 18 months	3	10
31 to 36 months	2	7
Over 37 months	24	80
Whether to participate in hospital work during the epidemic		
Yes	30	100
No	0	0
Whether to be exposed to a patient with suspected or confirmed COVID-19 diagnosis		
Yes	30	100
No	0	0

## Results

All FMEA team members initially identified different stages, processes, and corresponding potential failure modes in the CT examination process. This encompassed three stages, 10 processes, and 39 potential failure modes designed from the perspectives of Humans, Machine, Regulations, Environment, and objects.

Subsequently, all FMEA team members individually scored all potential failure modes from S, O, and D based on the CT examination process. The scoring scale ranged from 1 (*S*-insignificant/*O*-extremely unlikelihood/*D*-Absolutely sure) to 10 (*S*-catastrophic/*O*-inevitable/*D*-Absolutely abnormal). The individual scores assigned by the 30 team members in each index (*S*, *O*, and *D*) were integrated into a value. The median represented the score of this expert group for that particular index. The values of each index were then multiplied to derive the final integrated total score, which is RPN.

Finally, based on the RPN results, FMEA team members identified eight key risk factors, including “cleaning personnel does not wear masks normatively (*C*_32_) (RPN = 144),” “nurse does not select the vein well, resulting in extravasation of the peripheral vein for enhanced CT (*B*_25_) (RPN = 120),” “patient cannot find the CT room (*A*_13_) (RPN = 112.5),” “patient has obtained a CT request form but does not know the procedure (*A*_12_) (RPN = 110),” “patient is too unwell to continue with the CT scan (*B*_24_) (RPN = 97.5),” “auxiliary staff (or technician) does not have a good grasp of the sterilization and disinfection standards (*C*_21_) (RPN = 90),” “auxiliary staff (or technician) does not sterilize the CT machine thoroughly (*C*_22_) (RPN = 90),” and “cleaning personnel lacks of knowledge of COVID-19 prevention and control (*C*_33_) (RPN = 90)”. Furthermore, four key potential infection processes are summarized, namely “CT request form (*A*_1_),” “during the scan of CT patient (*B*_2_),” “CT room and objects disposal (*C*_2_),” and “medical waste (garbage) disposal (*C*_3_). Detailed FMEA analysis results of three stages, 10 processes and 39 potential failure modes in CT detection process are shown in Table 2.

**Table 2:** Risk analysis and RPN of the hospital radiology department

Stage	Processes	Element	Potential failure modes	Severity (S)	Occurrence (O)	Detection (D)	RPN	Rank
Before starting CT (*A*)	CT request form (*A*_1_)	Human	The CT request form does not meet the relevant norm, such as incomplete signs and symptoms (*A*_11_)	5.00	4.00	3.50	70	15
			Patient has obtained a CT request form but does not know the procedure (*A*_12_)	5.50	5.00	4.00	110	4
			Patient cannot find the CT room (*A*_13_)	5.00	5.00	4.50	112.5	3
		Machine	Examination for multiple body parts billed on the same CT request form (A_14_)	3.00	5.00	2.00	30	31
		Regulations	Excessive CT for COVID-19 Patients (*A*_15_)	3.50	3.00	4.50	47.25	21
			Priority of CT for emergency patients is not strictly implemented (conflicts with CT for outpatients) (*A*_16_)	5.00	4.00	4.00	80	12
	Patient identification (*A*_2_)	Human	Before CT, technician fails to input patient information correctly (*A*_21_)	9.00	2.00	2.00	36	25
			Before CT, technician fails to check the patient identification information normatively (*A*_22_)	7.50	2.00	2.00	30	31
			Before CT, technician fails to check the CT examination site correctly (*A*_23_)	8.50	2.00	2.00	34	26
			Before CT, technician fails to check patient’s identity and wristband information correctly (*A*_24_)	8.00	2.00	2.00	32	27
		Machine	Barcode gun malfunctions during information verification (*A*_25_)	3.00	2.00	2.00	12	39
	Technician entering the operating room (*A*_3_)	Human	Technician fails to wear N95 mask normatively (*A*_31_)	8.00	1.00	3.00	24	36
			Technician fails to wear isolation gowns normatively (*A*_32_)	7.00	2.00	2.00	28	35
			Technician lacks hand disinfection knowledge or fails to finish hand disinfection normatively (*A*_33_)	8.00	2.00	4.00	64	17
		object	Insufficient management of the reserve of infection protection materials, such as N95 masks, isolation gowns, and hand disinfectants (*A*_34_)	8.00	2.00	2.00	32	27
		Environment	The buffer zone between the CT operation room and CT room (changing isolation gowns) does not meet the requirements of prevention and control of COVID-19, including national regulations or hospital standards (*A*_35_)	9.00	1.00	2.00	18	38
During CT (*B*)	Before the CT patient’s boarding (*B*_1_)	Human	Patient does not remove metal objects (images may have artifacts) (*B*_11_)	6.00	3.00	2.50	45	22
		object	Life-saving medical equipment may affect CT scan, such as a large transfer bed (*B*_12_)	5.00	2.00	2.00	20	37
	During the scan of CT patient (*B*_2_)	Human	Poor respiratory coordination of patient(*B*_21_)	5.00	5.00	3.50	87.5	9
			Incorrect patient position (*B*_22_)	5.00	2.00	3.00	30	31
			Patient gets into bed difficulty (*B*_23_)	4.00	4.00	3.00	48	20
			Patient is too unwell to continue with the CT scan (*B*_24_)	6.50	3.00	5.00	97.5	5
			Nurse does not select the vein well, resulting in extravasation of the peripheral vein for enhanced CT (*B*_25_)	8.00	3.00	5.00	120	2
	The process of CT patient getting off the machine (*B*_3_)	Human	Patient gets off the machine too quickly or has difficulty (*B*_31_)	5.00	2.50	2.50	31.25	30
		object	CT machine is too high or not exiting, resulting in machine failure (*B*_32_)	7.50	2.00	2.00	30	31
	Common potential failure modes(*B*_4_)	Human	Technician fails to finish hand hygiene normatively (*B*_41_)	6.00	3.00	4.00	72	14
			Technician fails to communicate with the patient effectively (*B*_42_)	5.00	3.00	4.00	60	19
			Patient fails to wear the mask normatively (*B*_43_)	5.50	5.00	3.00	82.5	11
After CT (*C*)	The process of patient getting off CT room (*C*_1_)	Human	Patient forgets belongings (*C*_11_)	3.00	3.00	4.50	40.5	23
			Patient enquires about the time of output of the CT report (*C*_12_)	2.00	6.50	3.00	39	24
	CT room and objects disposal (*C*_2_)	Human	Auxiliary staff (or technician) does not have a good grasp of the sterilization and disinfection standards (*C*_21_)	6.00	3.00	5.00	90	6
			Auxiliary staff (or technician) does not sterilize the CT machine thoroughly (*C*_22_)	6.00	3.00	5.00	90	6
			Auxiliary personnel (or technician) does not sterilize the air in the CT room thoroughly (*C*_23_)	6.50	3.00	4.00	78	13
			Auxiliary staff (or technician) does not sterilize the X-ray protective clothing thoroughly, such as lead cloth, lead gown, lead bib, etc. (*C*_24_)	7.00	3.00	4.00	84	10
		Regulations	The COVID-19 prevention and control system of the CT room does not meet relevant standards (including environment, machines, etc.), including national regulations or hospital standards (*C*_25_).	8.00	2.00	2.00	32	27
	Medical waste (garbage) disposal (*C*_3_)	Human	Cleaning personnel does not handle medical waste normatively (*C*_31_)	7.50	3.00	3.00	67.5	16
			Cleaning personnel does not wear masks normatively (*C*_32_)	8.00	4.00	4.50	144	1
			Cleaning personnel lacks of knowledge of COVID-19 prevention and control (*C*_33_)	7.50	4.00	3.00	90	6
		Regulations	The infection prevention and control system of medical waste in CT rooms is imperfect, which should meet the national regulations or hospital standards (*C*_34_).	8.00	2.00	4.00	64	17

Note:

Severity (*S*): 1 is insignificant, and 10 is catastrophic

Occurrence (*O*): 1 is extremely unlikelihood, and 10 is inevitable

Detection (*D*): 1 is absolutely sure, and 10 is absolutely abnormal

## Discussion

### Clinical significance

The FMEA results of the case hospital identified eight key risk factors and four key potential corresponding essential prevention processes. The corresponding key risk factors are discussed in the following sequence:

For the “CT request form (*A*_1_)” process, “patient has obtained a CT request form but does not know the procedure (*A*_12_) (*RPN* = 110)” and “patient cannot find the CT room (*A*_13_) (*RPN* = 112.5)” are key risk factors. These issues are part of the patient access process within the case hospital. Under normal circumstances (in the absence of infectious disease), the patient could seek guidance from nurses, technicians, or others to complete the CT. However, the risk of nosocomial infections will increase owing to unnecessary contact during COVID-19 [26]. In the process of outpatient service delivery, formulating a service flowchart can streamline patient inquiries, avoid repetition in interpretation by hospital staff, improve the efficiency of hospital service delivery and patient service utilization, and ultimately improve the patient’s medical experience. The flowchart is often used as a simple and effective tool in formulating hospital service processes [27]. Therefore, the establishment of patient-centered CT flowcharts is recommended to enhance CT resource utilization and contribute to an improved patient experience.

For the “during the scan of CT patient (*B*_2_)” process, “nurse does not select the vein well, resulting in extravasation of the peripheral vein for enhanced CT (*B*_25_) (*RPN* = 120)” and “patient is too unwell to continue with the CT scan (*B*_24_) (*RPN* = 97.5)” are key risk factors. The selection of the injection vein is crucial in mitigating extravasation risk; an inappropriate choice of vein can lead to extravasation [28]. Concurrently, patients may experience sudden discomfort during the CT, such as local/systemic allergic reactions to the contrast agent [29]. In both cases, the patient is unable to complete the CT successfully, necessitating a repeat scan or rescheduling, which increases the patient’s usage frequency of the CT room and the frequency of doctor-patient contact, thereby increasing the risk of nosocomial COVID-19 infection [26]. Therefore, nurses should refine their proficiency in handling the indwelling needle and venous access. Simultaneously, physicians should improve the assessment of patients’ physical conditions before CT examination to reduce the possibility of patient discomfort during CT.

Concerning the process designated as “CT room and objects disposal (*C*_2_)” the key risk factors encompass “auxiliary staff (or technician) does not have a good grasp of the sterilization and disinfection standards (*C*_21_) (*RPN* = 90)” and “auxiliary staff (or technician) does not sterilize the CT machine thoroughly (*C*_22_) (*RPN* = 90)”. These factors may be because of inadequate training, insufficient knowledge of disinfection standards and specifications, and lack of effective supervision. The disinfection of equipment rooms and objects is a step in preventing and controlling nosocomial COVID-19 infection [2, 8] and requires appropriate attention. In COVID-19 prevention and control, it becomes imperative to elucidate and standardize the infection prevention and control knowledge and site disinfection. This entails strengthening the training and supervision of relevant personnel to ensure better implementation of infection control measures during the diagnostic radiological examination of COVID-19 cases [2].

Regarding the “medical waste (garbage) disposal (*C*_3_)” process, “cleaning personnel does not wear masks normatively (*C*_32_) (*RPN* = 144)” and “cleaning personnel lacks of knowledge of COVID-19 prevention and control (*C*_33_) (*RPN* = 90)” are key risk factors. The failure of cleaning staff to wear masks according to the norm scores the highest among all risk factors. Proper wearing of masks and other protective equipment constitutes the paramount measure in controlling the spread of COVID-19 [30]. A significant portion of COVID-19 transmissions can be attributed to improper mask-wearing and other protective equipment. Notably, cleaning staff are non-medical professionals, and their awareness of infectious disease prevention and control is much lower than that of healthcare workers. Their limited understanding and compliance with protective equipment usage and a potential lack of vigilance for disease would put non-frontline healthcare workers at higher risk of infection [30, 31]. Cleaning staff are responsible for the daily cleaning and disinfection of healthcare facilities and play an important role in preventing and controlling nosocomial COVID-19 infections [32]. Therefore, it is imperative to prioritize the training and supervision of cleaning staff in managing nosocomial COVID-19 infection.

### Practical applications

Based on the aforementioned FMEA results, the hospital management team can provide the following recommendations to control and improve the management of key risk factors during CT examinations.

For “cleaning personnel does not wear masks normatively (*C*_32_) (*RPN* = 144)”, the following improvement measures are suggested. First, address the issue of radiology cleaning staff. Subsequently, implement homogenization training and assessment [33]. Finally, the assessment results are linked with individual salary performance.

Regarding “nurse does not select the vein well, resulting in extravasation of the peripheral vein for enhanced CT (*B*_25_) (*RPN* = 120)”, the following improvement measures are proposed. First, the cephalic vein is the first choice for enhanced CT. For general enhancement or arterial series, appropriate indwelling needles should be used respectively. Then, for patients with poor venous condition, please consult the intravenous treatment team in the hospital under ultrasound guidance and perform difficult punctures. Finally, during CT, the patient should hold a venous alarm to call the nurse when the pain is uncomfortable, and the nurse can intervene early or in time to prevent venous extravasation [34].

Regarding “patient cannot find the CT room (*A*_13_) (*RPN* = 112.5)”, the following improvement measures are suggested. First, if it is an emergency patient, the hospital can increase the guiding staff in the emergency department area to assist the patient in navigating to the CT room. Subsequently, within the radiology department’s CT area, the hospital can deploy volunteers to enhance the accessibility of patients’ inquiries. Finally, add appropriate guidance signs from the patient’s point of view to increase patient autonomy, such as ground and wall.

For “patient has obtained a CT request form but does not know the procedure (*A*_12_) (*RPN* = 110)”, the following improvement measures are suggested. First, consider and modify the display mode of the original guide sheet from the patient’s point of view, and add the notification action of the CT examination process. Subsequently, the CT examination process video is added to the electronic education video of the radiology department. Finally, the video of the CT examination process is played in a continuous rolling way.

Addressing “patient is too unwell to continue with the CT scan (*B*_24_) (*RPN* = 97.5)”, the following improvement measures are recommended. Before the examination, CT technicians should initially inquire about patients’ medical history and allergies they might have. For unconscious patients, family members or medical personnel must be required to accompany them throughout the examination. In addition, communicate with patients at any time. If patients feel unwell, wave and call CT technicians and nursing staff in time.

Concerning “auxiliary staff (or technician) does not have a good grasp of the sterilization and disinfection standards (*C*_21_) (*RPN* = 90)”, the following improvement measures are proposed. Radiology assistants and technicians need a fixed time for training and assessment after the end to validate the training effectiveness [35]. During the epidemic period, daily inspections pertaining to COVID-19 are conducted in the department, ensuring stringent compliance with all disinfection standards.

For “auxiliary staff (or technician) does not sterilize the CT machine thoroughly (*C*_22_) (*RPN* = 90)”, the following improvement measures are suggested. First, the CT indoor air disinfection machine is opened regularly, four times a day, with an interval of 6 h each time [36]. Furthermore, use disposable sheets to ensure every patient uses the cleanest sheets. Subsequently, after daily inspections, disinfect the computer room for 1 h with an ultraviolet lamp [37]. Finally, the CT machine tool and air disinfection machine should be maintained regularly to ensure that the environment disinfection of the CT room is qualified.

Addressing “cleaning personnel lacks of knowledge of COVID-19 prevention and control (*C*_33_) (*RPN* = 90)”, the following improvement measures are recommended. First, establish fixed positions for the cleaning staff within the CT room. Subsequently, from the point of view of cleaning staff, provide training programs and implement assessment in a fixed and continuous manner. Finally, implement effective supervision and link it with performance. Crucially, ensure that the cleaning staff understands the principles and norms of COVID-19 infection prevention and control, emphasizing that implementation is the key to prevention.

### Limitations

First, it is worth acknowledging that the CT examination process and corresponding potential failure modes may differ slightly from hospital to hospital. Secondly, the participants in this study adopt purpose sampling, which may lead to sampling deviation. On the contrary, the lack of comparative analyses of FMEA with other risk assessment methods is one of the limitations of this study. Finally, FMEA results only show the current investigation status of the case hospital at that time and should not infer the subsequent time point. Simultaneously, the results should not be inferred from other hospitals.

## Conclusion

The findings of this study indicate the potential presence of key risk factors in the following procedures: (a) Patients might encounter difficulties locating the CT room before examination or do not understand the standard procedures of CT examination; (b) During the examination, nurses could select inappropriate veins, resulting in peripheral vein extravasation, and patients may struggle to adhere with the standard scanning procedures; (c) After examination, technicians (or auxiliary personnel) fail to carry out terminal disinfection treatment according to COVDID-19 standard prevention and control requirements. These problems could be preemptively addressed through good training and periodic retraining of the involved personnel. In addition, hospitals can publicize the precautions in the examination through various channels, which can reduce the incidence of failure in CT examination and make patients, medical staff, technicians, and auxiliary personnel actively participate and cooperate well. Finally, cleaning services in hospitals are usually outsourced, and their educational background is usually low. Therefore, in training and communication, it is crucial to provide a series of targeted training programs from a perspective that they can understand.

Radiology is crucial in preventing and controlling COVID-19, and CT examination is one of the keys to preventing and controlling infection. For suspected or confirmed COVID-19 patients, early examination, diagnosis, and isolation are imperative to mitigate hospital-acquired infections and potential systemic disruptions (such as the breakdown of the hospital medical system). Simultaneously, the CT examination need of other patients should also be prioritized.

FMEA risk assessment can identify potential risks during CT examination during COVID-19. Hospital decision-makers and managers can improve the occupational protection awareness of medical and cleaning staff in the radiology department through corresponding prevention planning and measures. In addition, this kind of research with practical application will help medical staff establish a risk management and control model of the CT examination process for major infectious epidemics in hospitals from the practical experience during the COVID-19 epidemic. The model can effectively identify the key infection prevention processes and key risk factors and better protect the safety of medical staff and patients. This is of great research significance in the face of potential major infectious epidemics in the future.

## Data Availability

The original contributions presented in the study are included in the article materials, further inquiries can be directed to the corresponding authors.
